# Intravenous immunoglobulin resistance in Kawasaki disease patients: prediction using clinical data

**DOI:** 10.1038/s41390-023-02519-z

**Published:** 2023-02-16

**Authors:** Jonathan Y. Lam, Min-Seob Song, Gi-Beom Kim, Chisato Shimizu, Emelia Bainto, Adriana H. Tremoulet, Shamim Nemati, Jane C. Burns

**Affiliations:** 1 Department of Biomedical Informatics, University of California San Diego, La Jolla, CA USA; 2 Department of Pediatrics, Haeundae Paik Hospital, Inje University, Busan, South Korea; 3 Department of Pediatrics, Seoul National University Children’s Hospital, Seoul National University College of Medicine, Seoul, South Korea; 4 Department of Pediatrics, Rady Children’s Hospital and University of California San Diego, San Diego, CA USA

## Abstract

**Background:**

About 10–20% of Kawasaki disease (KD) patients are resistant to the initial infusion of intravenous immunoglobin (IVIG). The aim of this study was to assess whether IVIG resistance in KD patients could be predicted using standard clinical and laboratory features.

**Methods:**

Data were from two cohorts: a Korean cohort of 7101 KD patients from 2015 to 2017 and a cohort of 649 KD patients from San Diego enrolled from 1998 to 2021. Features included laboratory values, the worst *Z*-score from the initial echocardiogram or during hospitalization, and the five clinical KD signs at presentation.

**Results:**

Five machine learning models achieved a maximum median AUC of 0.711 [IQR: 0.706–0.72] in the Korean cohort and 0.696 [IQR: 0.609–0.722] in the San Diego cohort during stratified 10-fold cross-validation using significant laboratory features identified from univariate analysis. Adding the *Z*-score, KD clinical signs, or both did not considerably improve the median AUC in either cohort.

**Conclusions:**

Using commonly measured clinical laboratory data alone or in conjunction with echocardiographic findings and clinical features is not sufficient to predict IVIG resistance. Further attempts to predict IVIG resistance will need to incorporate additional data such as transcriptomics, proteomics, and genetics to achieve meaningful predictive utility.

**Impact:**

- We demonstrated that laboratory, echocardiographic, and clinical findings cannot predict intravenous immunoglobin (IVIG) resistance to a clinically meaningful extent using machine learning in a homogenous Asian or ethnically diverse population of patients with Kawasaki disease (KD).
- Visualizing these features using uniform manifold approximation and projection (UMAP) is an important step to evaluate predictive utility in a qualitative manner.
- Further attempts to predict IVIG resistance in KD patients will need to incorporate novel biomarkers or other specialized features such as genetic differences or transcriptomics to be clinically useful.

## Introduction

Kawasaki disease (KD) is an acute pediatric disorder of unknown cause characterized by coronary artery vasculitis and is the leading cause of acquired heart disease in children from developed countries.^1^ The standard treatment for KD is a single dose of intravenous immunoglobin (IVIG). However, 10–20% of KD patients are resistant to the first infusion of IVIG and are at increased risk for coronary artery aneurysms.^2,3^ Early identification of these patients might improve patient outcomes through more timely treatment with additional anti-inflammatory agents.^4^

There have been numerous diagnostic risk scores developed to predict IVIG resistance in KD patients, most notably the Kobayashi score in Japan.^5^ This score, along with other Japanese scores such as the Egami score^6^ and Sano score,^7^ have been used in Japan to stratify KD patients into predicted IVIG non-responders and responders,^8^ but have shown poor generalizability in non-Japanese populations.^9–12^ Despite the lack of demonstrated clinical utility for non-Japanese populations, IVIG resistance risk scores continue to be developed using a combination of demographics, laboratory features, echocardiographic findings, and/or clinical features.^12^ Stratifying patients with high risk scores and pre-emptively administering additional therapies in responders who were predicted to be IVIG non-responders may lead to potential adverse events.^8^ Alternatively, the low sensitivity reported in several scores results in high-risk patients being wrongly predicted as low risk.^9,11,12^

Without a proper assessment of the predictive ability of these features, it seems inappropriate to continue developing risk scores for IVIG resistance. Traditionally, features are selected based on significant differences between responders and non-responders and a composite risk score or machine learning model is constructed to discriminate between these two populations based on a set threshold. This approach is reliant on the identified feature set having sufficient predictive ability as assessed through validation on an independent cohort in terms of time or location. When studies do not include independent validation, it is unknown whether the proposed model is overfitting to the training data.^5–7,13,14^ In studies with independent validation, there are instances where the reported performance metrics are likely too low for clinical use and would fail to improve patient outcomes if an impact assessment were to be conducted.^3,15^ Before constructing any model, it is useful to visualize the data to determine if the feature set can discriminate responders from non-responders. One visualization technique is uniform manifold approximation and projection (UMAP), which constructs a high-dimension graph based on input data and then projects the graph into lower dimensions.^16^ This enables the visualization of numerous features in a two-dimensional scatter plot, which can be used to qualitatively assess a dataset based on the separation between individual samples or clusters using pre-defined labels. In this study, our aim was to assess whether laboratory, echocardiographic, and/or clinical findings could be used to predict IVIG resistance in KD patients using UMAP and common machine learning algorithms. Through this approach, we qualitatively evaluated whether the features could discriminate responders from non-responders and leveraged the ability of machine learning algorithms to analyze complex sets of predictors with greater accuracy than rule-based models in clinical applications such as discharge diagnoses.^17^

## Methods

### Korean study population

A total of 7101 patients diagnosed with KD in Korea (“Korean cohort”) with complete data were retrospectively identified from a questionnaire survey under the guidance of the Korean Society of Kawasaki Disease to investigate epidemiologic features of KD.^18^ Data were collected on patients with acute-phase KD who were treated between January 1, 2015 and December 31, 2017. Laboratory and clinical measurements were acquired at the time of initial evaluation prior to IVIG administration. The laboratory variables were white blood count (WBC), neutrophil percentage, hemoglobin, platelet count, C-reactive protein (CRP), erythrocyte sedimentation rate (ESR), total protein, albumin, aspartate aminotransferase (AST), alanine aminotransferase (ALT), total bilirubin, and sodium. Other continuous variables were age, weight, height, illness days, and the worst *Z*-score from echocardiograms during initial hospitalization (worst *Z*-score during hospitalization). Binary variables were sex, rash, conjunctival injection, oral changes, extremity changes, cervical lymphadenopathy, and sterile pyuria according to the American Heart Association definition if applicable.^1^

### San Diego study population

A total of 649 patients diagnosed with KD at Rady Children’s Hospital in San Diego, California from January 1, 1998 to July 12, 2021 with complete data were identified from a REDCap database at the Kawasaki Disease Research Center in the University of California San Diego (“San Diego cohort”). This cohort was comprised of patients who were initially treated with only IVIG and excluded those (*n* = 258) who received intensification of initial therapy because of coronary artery dilation by echocardiography. Laboratory variables were white blood count with manual differential (total white blood count, neutrophils, bands, lymphocytes, atypical lymphocytes, monocytes, and eosinophils), platelets, hemoglobin, ESR, ALT, CRP, albumin, and gamma-glutamyl transferase (GGT). Other continuous variables included age, weight, height, illness days, and worst *Z*-score from the initial echocardiogram at the time of diagnosis (worst initial *Z*-score). Binary variables included sex, rash, red eyes, oral changes, extremity changes, and cervical lymphadenopathy. All laboratory and clinical features were collected before the first IVIG infusion. Echocardiography was performed within the first 24 h after admission.

All KD patients met the case definition of the American Heart Association for either complete or incomplete KD.^1^ Informed consent was not required for the Korean cohort but obtained for patients in the San Diego cohort under a study approved by the University of California San Diego Institutional Review Board. Patients in both cohorts were considered IVIG-resistant if they had persistent or recrudescent fever (>38 °C) 36 h or more after the completion of IVIG infusion. The *Z*-score was calculated using the Dallaire equation on echocardiogram findings for the left anterior descending artery and right coronary artery.^19^

### Model development

For each cohort, logistic regression, random forest, Naive Bayes classifier, gradient boosting machine, and feedforward neural network models were fit to the data. Multivariate logistic regression has been the most popular method for developing IVIG resistance risk scores.^2,5,20^ The other models were chosen based on prior use in IVIG resistance risk score development (random forest, gradient boosting machine),^15^ comparable performance with other models (Naive Bayes classifier), or ability to account for higher level interactions between features (feedforward neural network).^17^ Models were developed using scikit-learn^21^ (v1.0.2) or TensorFlow^22^ (v2.9.1) for the feedforward neural networks. Data were split 80:20 into a training and test set when performing stratified 10-fold cross validation on predictive models in each cohort independently. Performance of the models was evaluated using area under the receiver operating characteristic curve (AUC), sensitivity (SEN), specificity (SPC), area under the precision recall curve (AUPRC), positive predictive value (PPV), negative predictive value (NPV), and accuracy (ACC) calculated at a minimum 93% specificity for responders. For temporal validation, test sets were set in the Korean cohort by year. In the San Diego cohort, test sets were established by splitting the data into five chronological bins with similar numbers of patients. When validating the cohorts against each other, data were filtered using the common features in both cohorts before training models on the entirety of one cohort and evaluating model performance on the other cohort. All data were normalized by subtracting the mean and dividing by the standard deviation within each feature using the respective values from the training set.

### UMAP embeddings

We used uniform manifold approximation and projection (UMAP) to reduce the dimensionality of normalized data and produce embeddings in two dimensions.^16^ Default parameters from the umap-learn Python package (v0.5.3) were used to create the embeddings.

### Statistical analysis

Chi-square test was used to compare categorical variables and the Mann–Whitney *U*-test used to compare continuous variables. A *p* value < 0.05 was considered significant. All statistical analyses were performed in Python 3.9 using the Scipy library (v1.7.3).

## Results

We identified significant features between IVIG-responsive and IVIG-resistant patients in both cohorts to use for downstream analysis (Supplementary Tables S1 and S2). The resistance rate for the first IVIG treatment was 17.5% (1241/7101) in the Korean cohort and 19.9% (129/649) in the San Diego cohort. While the Korean cohort consisted of a homogenous population of ethnic Koreans, the San Diego cohort consisted of a mixed population self-reported by parents as follows: 35.3% (229/649) Hispanic, 25.3% (164/649) White, 16.6% (108/649) Asian, 2.9% (19/649) Black/African American, and 19.9% (129/649) Mixed/Other.

### UMAP embedding

**Fig. 1:**
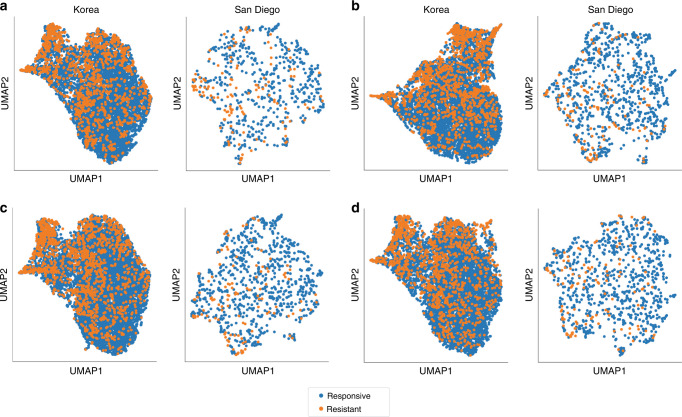
UMAP embeddings of KD patients for the Korean and San Diego cohorts labeled by IVIG resistance. **a** Normalized significant laboratory features only, **b** normalized significant laboratory features and echocardiographic findings (worst *Z*-score), **c** normalized significant laboratory features and significant classical KD signs, and **d** normalized significant laboratory features, echocardiographic findings (worst *Z*-score), and significant classical KD signs.

We first conducted low-dimensional visualizations to assess patient separation using significant laboratory values, the worst *Z*-score, and the KD clinical signs as features (Fig. 1). There was no clear separation of the IVIG-responders from the IVIG-resistant population using significant laboratory features in both cohorts. The embeddings from combining the laboratory features with the *Z*-score or the KD clinical signs also failed to clearly separate the two populations in either cohort. Finally, combining laboratory features with the *Z*-score and significant clinical signs did not result in clear separation. In addition, overlaying the presence of coronary artery lesions as defined by a worst *Z*-score ≥2.5 resulted in a heterogenous distribution using the same combinations of features (Supplementary Fig. 1).

### Internal validation

**Fig. 2:**
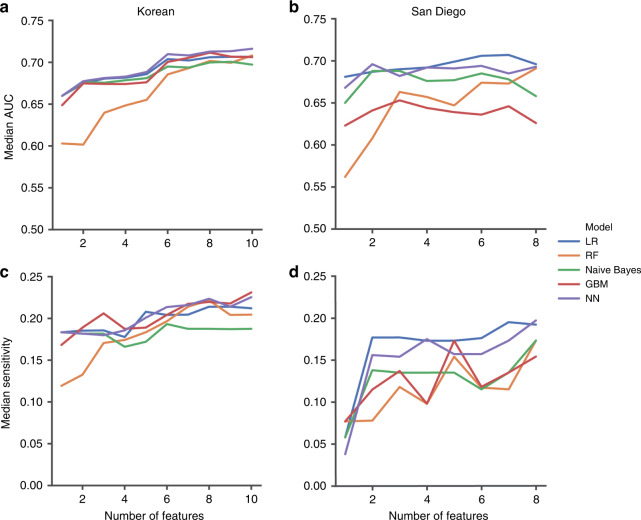
Performance of models trained on feature sets defined through recursive feature elimination. **a** Median AUC from the Korean cohort, **b** median AUC from the San Diego cohort, **c** sensitivity from the Korean cohort, and **d** sensitivity from the San Diego cohort. LR logistic regression, RF random forest, GBM gradient boosting machine, NN neural network.

We next trained five different types of machine learning models on the data (logistic regression (LR), random forest (RF), Naive Bayes classifier (Naive Bayes), gradient boosting machine (GBM), and feedforward neural network (NN)) and achieved a maximum median AUC of 0.711 [IQR: 0.706–0.72] during 10-fold cross-validation within the Korean cohort (Table 1). The same models fit to the San Diego cohort showed similar performance with a maximum median AUC of 0.696 [IQR: 0.609–0.722]. With thresholds set to detect a minimum of 93% of the responders, no model achieved a median sensitivity for the non-responders higher than 21.6%. Recursive feature elimination within each cohort by sequentially removing features one at a time from the feature set based on significance did not identify a parsimonious set of features with substantially improved performance (Fig. 2). In addition to randomly splitting the data, we performed temporal validation within each cohort. Korean KD patients hospitalized in 2016 and San Diego KD patients enrolled from 2008 to 2010 had the highest median AUC at 0.729 and 0.742, respectively (Supplementary Table S3). Using only KD patients with 5 days or less of illness did not improve performance in terms of median AUC (Supplementary Table S4). Adding the worst *Z*-score or the classical KD signs also did not improve performance. Although the worst *Z*-score for the Korean cohort may be from an echocardiogram after IVIG treatment, the poor performance observed in the San Diego cohort when including the worst *Z*-score from the initial echocardiogram demonstrates the lack of utility of echocardiographic findings for predicting IVIG resistance. Combining laboratory, echocardiographic, and clinical data did not considerably increase the maximum median AUC in comparison with the models trained using laboratory data only in the Korean (0.724 vs. 0.711) and San Diego (0.727 vs. 0.696) cohort. We repeated the combination of laboratory, echocardiographic, and clinical data in patients without dilated coronary arteries by excluding patients with a worst *Z*-score ≥2.5 and found no improvement in the maximum median AUC with either the Korean (0.718 vs. 0.724) or San Diego (0.708 vs. 0.727) cohort.

**Table 1:** Tenfold stratified cross validation performance metrics for the Korean and San Diego cohorts using laboratory findings.

	AUC	SEN	SPC	AUPRC	PPV	NPV	ACC
Korea							
LR	0.698 (0.695–0.707)	0.215 (0.166–0.265)	0.948 (0.948–0.949)	0.367 (0.35–0.373)	0.469 (0.427–0.511)	0.851 (0.843–0.858)	0.82 (0.818–0.824)
RF	0.696 (0.685–0.702)	0.195 (0.17–0.221)	0.949 (0.949–0.95)	0.357 (0.352–0.367)	0.466 (0.434–0.498)	0.848 (0.845–0.852)	0.82 (0.818–0.822)
Naive Bayes	0.697 (0.688–0.707)	0.181 (0.151–0.212)	0.949 (0.947–0.95)	0.333 (0.327–0.339)	0.435 (0.398–0.471)	0.846 (0.841–0.851)	0.816 (0.815–0.82)
GBM	0.704 (0.699–0.717)	0.214 (0.19–0.237)	0.95 (0.949–0.95)	0.368 (0.366–0.376)	0.475 (0.444–0.507)	0.851 (0.848–0.854)	0.821 (0.819–0.824)
NN	0.711 (0.706–0.72)	0.216 (0.188–0.243)	0.949 (0.947–0.952)	0.407 (0.382–0.42)	0.476 (0.437–0.514)	0.851 (0.847–0.856)	0.821 (0.818–0.823)
San Diego							
LR	0.696 (0.609–0.722)	0.192 (0.046–0.338)	0.942 (0.933–0.942)	0.358 (0.305–0.462)	0.5 (0.277–0.723)	0.825 (0.797–0.853)	0.8 (0.785–0.819)
RF	0.691 (0.604–0.707)	0.173 (0.109–0.237)	0.942 (0.942–0.942)	0.328 (0.276–0.381)	0.438 (0.278–0.597)	0.822 (0.811–0.832)	0.795 (0.786–0.806)
Naive Bayes	0.658 (0.596–0.707)	0.173 (0.046–0.3)	0.942 (0.925–0.942)	0.32 (0.267–0.38)	0.444 (0.274–0.615)	0.82 (0.791–0.849)	0.792 (0.779–0.8)
GBM	0.626 (0.58–0.686)	0.154 (0.042–0.266)	0.942 (0.923–0.942)	0.268 (0.25–0.362)	0.323 (0.124–0.522)	0.817 (0.808–0.826)	0.776 (0.768–0.79)
NN	0.693 (0.636–0.712)	0.197 (0.053–0.341)	0.933 (0.923–0.942)	0.364 (0.324–0.473)	0.41 (0.197–0.623)	0.821 (0.795–0.847)	0.795 (0.776–0.819)

All values are reported as median (IQR).

*LR* logistic regression, *RF* random forest, *GBM* gradient boosting machine, *NN* neural network, *AUC* area under the receiver operating curve, *SEN* sensitivity, *SPC* specificity, *AUPRC* area under the precision recall curve, *PPV* positive predictive value, *NPV* negative predictive value, *ACC* accuracy.

### External validation

After conducting internal validation within each cohort, we developed predictive models by training models on one cohort and validating the model on the other cohort. The common laboratory features in both cohorts used for external validation were neutrophils, ALT, CRP, albumin, platelet count, ESR, and age-adjusted hemoglobin. Using the same types of models in internal validation, we found the median AUC to be <0.69 (Table 2). This poor validation performance is consistent with the results of other IVIG-resistance risk scores based on laboratory results applied to our cohorts. The Sano^6^ and Formosa^20^ scores had an AUC of 0.639 and 0.626 in the Korean cohort and an AUC of 0.628 and 0.517 in the San Diego cohort, respectively. The Kobayashi,^5^ Sano,^7^ and Yang^23^ scores had an AUC of 0.641, 0.641, and 0.626 in the Korean cohort and could not be evaluated in the San Diego cohort because of missing variables.

**Table 2:** External validation of models trained on one cohort and tested on the other cohort.

	AUC	SEN	SPC	AUPRC	PPV	NPV	ACC
Train Korea, validate San Diego							
LR	0.658	0.178	0.944	0.353	0.451	0.822	0.792
RF	0.653	0.178	0.95	0.318	0.469	0.823	0.797
Naive Bayes	0.621	0.202	0.94	0.303	0.456	0.826	0.794
GBM	0.65	0.14	0.948	0.321	0.4	0.816	0.787
NN	0.66	0.186	0.948	0.365	0.471	0.824	0.797
Train San Diego, validate Korea							
LR	0.685	0.176	0.95	0.318	0.428	0.845	0.815
RF	0.632	0.14	0.95	0.281	0.382	0.839	0.81
Naive Bayes	0.643	0.124	0.95	0.277	0.345	0.837	0.806
GBM	0.599	0.086	0.95	0.241	0.268	0.831	0.799
NN	0.685	0.177	0.948	0.323	0.421	0.845	0.814

*LR* logistic regression, *RF* random forest, *GBM* gradient boosting machine, *NN* neural network, *AUC* area under the receiver operating curve, *SEN* sensitivity, *SPC* specificity, *AUPRC* area under the precision recall curve, *PPV* positive predictive value, *NPV* negative predictive value, *ACC* accuracy.

## Discussion

In this study, we demonstrated that using common laboratory features alone or in conjunction with initial echocardiographic findings and clinical features cannot predict IVIG resistance in KD patients to a clinically meaningful extent in either an ethnically diverse or homogenous population. Although there is no clear definition for determining if a risk score or model has clinically meaningful utility, the low performance observed with numerous machine learning models in our study means that it is highly unlikely for any risk score constructed using these types of features to have sufficient predictive power to be clinically useful. Despite the abundance of IVIG resistance risk scores using similar features, none have been generalizable to external cohorts with racial or ethnogeographic differences.^9–12,24^ The major complication is the shift in data distribution or prevalence of features between cohorts despite similar demographics. For example, the prevalence of rash, conjunctival injection, oral changes, and extremity changes is higher in the San Diego cohort compared to the Korean cohort due to the patient population or clinical assessment. Laboratory results such as alanine aminotransferase have different distributions possibly due to how laboratory tests are manufactured in a particular country.

The purpose of an IVIG resistance risk score is to identify resistant patients preemptively to allow administration of additional therapies to reduce the risk of coronary artery abnormalities. IVIG risk scores are commonly used in Japan for deciding who should receive steroids or other adjunctive therapies. As with all treatments, the risks and benefits must be assessed. After excluding patients with initial abnormal echocardiograms, there is likely no benefit to IVIG responders in receiving additional therapies. Using the positive predictive values from the internal validation of the Korean cohort as an example, there would be approximately one false positive for every correctly identified IVIG-resistant patient. No model had a median sensitivity higher than 0.25 meaning that more than three of every four IVIG non-responders would be missed. This performance is not sufficient to warrant use of risk scores in the clinical workflow for KD patients.

Low-dimensional visualizations generated by UMAP are a powerful method for understanding patient populations. If the discrimination reported by various risk scores is true, generating UMAP embeddings using the corresponding features and overlaying the response to IVIG treatment should have clear separation between the two populations. Otherwise, the lack of separation as observed in our cohorts demonstrates that predicting IVIG resistance cannot be resolved using the selected features alone. For future studies, visualizing the patient population using this method is an important step in determining whether a set of features has adequate predictive utility.

Our study has several limitations. Data for San Diego cohort were prospectively collected, while the data for the Korean cohort were retrospectively collected via a questionnaire specifically designed to collect data for an IVIG-resistance scoring system. The compiled data does not contain several laboratory tests or values that are present in other risk scores such as aspartate aminotransferase (AST) and sodium in the San Diego cohort or the neutrophil-to-lymphocyte and platelet-to-lymphocyte ratios proposed by Kanai et al.^25^. It is unknown how the inclusion of these test results would affect the performance of the machine learning models. However, we have shown that the inclusion of AST and sodium in internal validation within the Korean cohort did not lead to good performance. Furthermore, the Kanai ratios are accounted for in the feedforward neural networks that have the ability to model complex relationships between features including ratios. We cannot exclude the possibility of misdiagnosis in the San Diego cohort during the coronavirus disease 2019 pandemic, but temporal validation showed poor performance in pre-pandemic validation cohorts. We also could not validate risk scores that required features missing from our cohorts including ferritin and procalcitonin because such features were not routinely ordered at the time of hospital admission.^14,26^

In conclusion, the model performances were not adequate to justify using only laboratory, echocardiographic, and/or clinical features to predict IVIG resistance. An acceptable model or risk score will need to incorporate novel biomarkers or other specialized features such as genetic differences or transcriptomics that can discriminate between IVIG resistant and responsive populations and demonstrate generalizable performance in an external cohort or during prospective validation with an improvement in patient outcomes through an impact study. Until then, IVIG resistance risk scores using laboratory, echocardiographic, and/or clinical features have little utility.

### Supplementary Information

Supplementary Information

## Data Availability

The datasets in the study are available from the corresponding author on reasonable request.
