# Supplementary material for: Intravenous immunoglobulin resistance in Kawasaki disease patients: prediction using clinical data
Source: Pediatr Res. 2023 Feb 16;95(3):692–7. doi: 10.1038/s41390-023-02519-z (PMC9934506; doi:10.1038/s41390-023-02519-z)
Supplement: Supplementary file 1 — Supplementary Information [file 41390_2023_2519_MOESM1_ESM.pdf]

Supplementary Information

Supplementary Figure 1: UMAP embeddings of KD patients for the Korean and San Diego cohorts using (A) normalized significant laboratory features, (B) normalized significant laboratory features and echocardiographic findings (worst Z-score), (C) normalized significant laboratory features and significant classical KD signs, and (D) normalized significant laboratory features, echocardiographic findings (worst Z-score), and significant classical KD signs labeled by presence of coronary artery lesions (CAL).

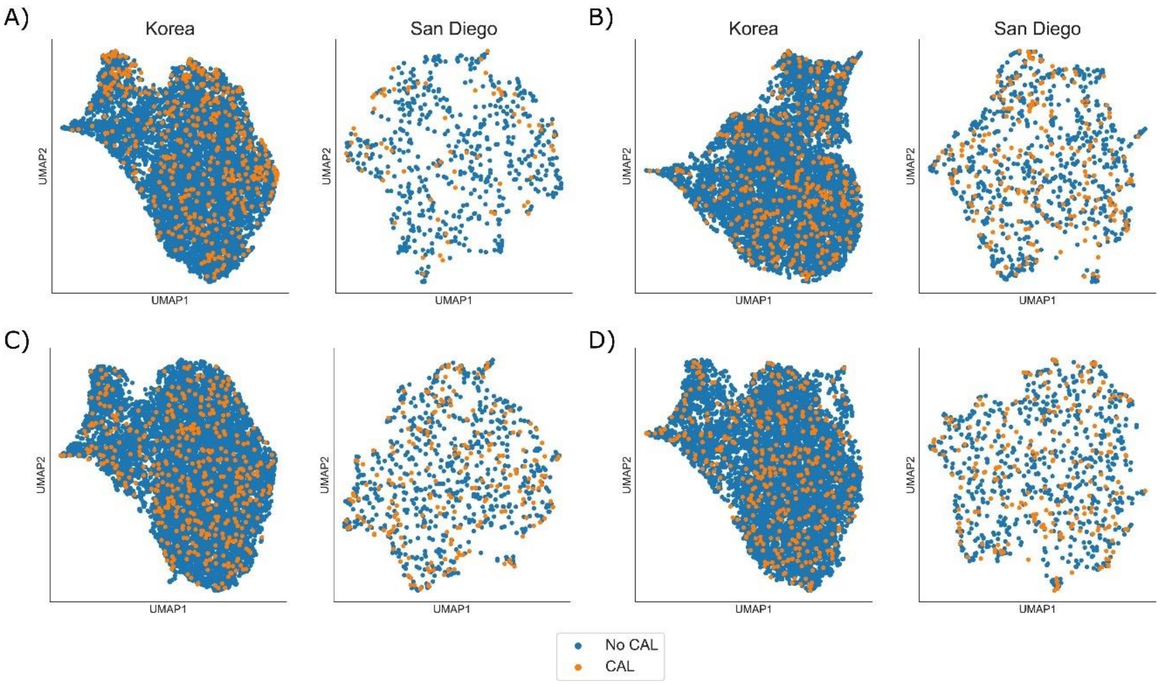

Supplementary Table S1: Clinical characteristics of the Korean cohort (n=7,101) in order of significance of the difference between responsive and resistant KD patients organized by four categories of variables. Values are median (IQR) for continuous variables and number (percentage) for binary variables.

	Responsive (n=5860)	Resistant (n=1241)	P value
**Demographic Variables**			
Illness Days	5.0 (4.0-6.0)	5.0 (4.0-5.0)	2.20E-12
Weight (kg)	13.0 (10.3-16.4)	13.9 (11.2-17.0)	1.51E-07
Age (months)	30.0 (14.0-48.0)	33.0 (19.0-51.0)	4.72E-07
Height (cm)	92.0 (79.0-102.7)	94.0 (83.0-104.4)	1.45E-06
Sex (male)	3353 (57.3%)	762 (61.8%)	4.21E-03
**Laboratory Results**			
Neutrophil (%)*	64.1 (52.1-74.6)	73.9 (61.9-82.5)	4.18E-67
Total Bilirubin	0.38 (0.26-0.55)	0.5 (0.3-1.19)	3.43E-53
Alanine Aminotransferase	25.0 (14.0-92.0)	60.0 (17.0-189.0)	5.20E-35
Aspartate Aminotransferase	35.0 (26.0-61.0)	48.0 (29.0-135.0)	2.55E-34
C-Reactive Protein	5.91 (3.28-10.0)	8.18 (4.73-12.8)	9.12E-34
Albumin	3.9 (3.7-4.2)	3.8 (3.4-4.1)	1.17E-21
Sodium	137.0 (135.0-138.0)	136.0 (134.0-138.0)	5.08E-20
Platelet Count	351.5 (290.0-420.0)	324.0 (264.0-392.0)	8.88E-17
Erythrocyte Sedimentation Rate	54.0 (37.0-73.0)	57.0 (39.0-76.0)	2.27E-03
Age-Adjusted Hemoglobin	-0.83 (-1.67-0.0)	-0.89 (-1.8--0.12)	4.28E-03
Total Protein	6.6 (6.2-7.0)	6.6 (6.1-7.0)	1.42E-01
White Blood Count	13.72 (10.77-17.19)	13.82 (10.68-17.7)	1.66E-01
**Clinical Variables**			
Rash	4460 (76.1%)	1029 (82.9%)	2.42E-07
Extremity Changes	3864 (65.9%)	907 (73.1%)	1.31E-06
Cervical Lymphadenopathy	3374 (57.6%)	806 (64.9%)	1.92E-06
Pyuria	2008 (34.3%)	509 (41.0%)	7.38E-06
Conjunctival Injection	5226 (89.2%)	1067 (86.0%)	1.49E-03
Oral Changes	4946 (84.4%)	1024 (82.5%)	1.08E-01
**Echocardiogram Findings**			
Worst Z-Score during hospitalization	0.29 (-0.28-0.97)	0.54 (-0.11-1.37)	1.43E-14

*Includes bands

Supplementary Table S2: Clinical characteristics of the San Diego cohort (n=649) in order of significance of the difference between responsive and resistant KD patients organized by four categories of variables. Values are median (IQR) for continuous variables and number (percentage) for binary variables.

	Responsive (n=520)	Resistant (n=129)	P value
**Demographic Variables**			
Illness Days	6.0 (4.0-7.0)	5.0 (4.0-6.0)	1.52E-03
Height (cm)	93.0 (81.0-108.0)	97.0 (85.0-109.1)	1.86E-01
Age (months)	2.75 (1.52-4.67)	2.86 (1.72-4.85)	2.45E-01
Weight (kg)	13.7 (10.68-18.02)	14.01 (11.4-18.9)	2.88E-01
Sex (male)	301 (57.9%)	78 (60.5%)	6.65E-01
**Laboratory Results**			
Alanine Aminotransferase	37.0 (22.0-107.25)	87.0 (39.0-151.0)	3.46E-09
Bands (%)	8.0 (3.0-16.0)	15.0 (7.0-28.0)	3.00E-08
Gamma-Glutamyl Transferase	35.5 (17.0-123.0)	90.0 (29.0-163.0)	2.42E-05
Lymphocytes (%)	21.0 (13.0-32.0)	16.0 (10.0-24.0)	2.63E-05
C-Reactive Protein	6.5 (3.8-14.6)	8.1 (5.2-18.8)	1.51E-03
Platelet Count	359.0 (279.75-453.25)	324.0 (249.0-420.0)	3.31E-03
Atypical Lymphocytes (%)	0.0 (0.0-2.0)	0.0 (0.0-1.0)	1.66E-02
Albumin	3.8 (3.5-4.2)	3.7 (3.4-4.0)	3.11E-02
Eosinophils	2.0 (1.0-4.0)	2.0 (0.0-4.0)	8.46E-02
Monocytes (%)	6.0 (4.0-8.0)	5.0 (3.0-7.0)	1.01E-01
Erythrocyte Sedimentation Rate	59.0 (38.0-75.0)	53.0 (37.0-66.0)	1.46E-01
White Blood Count	13.5 (10.4-16.9)	12.8 (10.5-16.3)	3.25E-01
Age-Normalized Hemoglobin	-1.33 (-2.17--0.44)	-1.33 (-2.17-0.0)	4.93E-01
Neutrophil (%)*	55.0 (44.0-66.0)	55.0 (40.0-66.0)	5.85E-01
**Clinical Variables**			
Extremity Changes	407 (78.3%)	114 (88.4%)	1.40E-02
Conjunctival Injection	480 (92.3%)	126 (97.7%)	4.59E-02
Oral Changes	489 (94.0%)	126 (97.7%)	1.50E-01
Rash	480 (92.3%)	123 (95.3%)	3.11E-01
Cervical Lymphadenopathy	179 (34.4%)	43 (33.3%)	8.97E-01
**Echocardiographic Findings**			
Worst initial Z-Score	1.39 (0.96-1.85)	1.47 (0.96-2.04)	2.62E-01

*Does not include bands

Supplementary Table S3: Temporal cross-validation AUC and sensitivity results for the Korean and San Diego cohorts using laboratory findings. Columns represent the years used for the test set. The threshold was set at 93% specificity. AUC: Area under the receiver operator characteristic curve, LR: logistic regression, RF: random forest, GBM: gradient boosting machine, NN: neural network

	San Diego					Korea		
**AUC**	**1998-2007**	**2008-2010**	**2011-2013**	**2014-2016**	**2017-2021**	**2015**	**2016**	**2017**
LR	0.649	0.72	0.725	0.638	0.662	0.715	0.709	0.682
RF	0.672	0.698	0.708	0.666	0.612	0.688	0.714	0.656
Naïve Bayes	0.638	0.725	0.683	0.651	0.698	0.704	0.715	0.669
GBM	0.653	0.692	0.665	0.628	0.585	0.719	0.715	0.672
NN	0.663	0.742	0.734	0.666	0.668	0.729	0.723	0.696
**Sensitivity**	**1998-2007**	**2008-2010**	**2011-2013**	**2014-2016**	**2017-2021**	**2015**	**2016**	**2017**
LR	0.125	0.36	0.267	0.259	0.133	0.217	0.255	0.204
RF	0.219	0.08	0.2	0.185	0.1	0.195	0.222	0.156
Naïve Bayes	0.031	0.28	0.333	0.222	0.1	0.152	0.227	0.166
GBM	0.062	0.04	0.067	0.148	0.033	0.217	0.214	0.19
NN	0.094	0.36	0.333	0.333	0.133	0.207	0.225	0.193

Supplementary Table S4: Median AUC (IQR) from 10-fold stratified cross validation for the Korean and San Diego cohorts using laboratory findings on patients who present with 5 days or less of fever (FEV), with echocardiographic findings (EF), with significant clinical findings (CF), and with echocardiographic and clinical findings (EF+CF). LR: logistic regression, RF: random forest, GBM: gradient boosting machine, NN: neural network

	**FEV**	**EF**	**CF**	**EF+CF**
Korea				
LR	0.706 (0.699-0.707)	0.705 (0.703-0.717)	0.709 (0.706-0.716)	0.716 (0.712-0.724)
RF	0.698 (0.689-0.705)	0.708 (0.693-0.712)	0.7 (0.689-0.713)	0.714 (0.709-0.723)
Naïve Bayes	0.696 (0.692-0.699)	0.705 (0.699-0.712)	0.706 (0.697-0.714)	0.709 (0.707-0.717)
GBM	0.707 (0.701-0.718)	0.712 (0.708-0.719)	0.707 (0.705-0.723)	0.72 (0.712-0.726)
NN	0.711 (0.706-0.718)	0.719 (0.71-0.722)	0.719 (0.716-0.728)	0.724 (0.719-0.732)
San Diego				
LR	0.645 (0.617-0.698)	0.697 (0.606-0.724)	0.702 (0.635-0.731)	0.704 (0.629-0.729)
RF	0.7 (0.675-0.714)	0.656 (0.613-0.675)	0.679 (0.626-0.703)	0.674 (0.617-0.692)
Naïve Bayes	0.619 (0.601-0.727)	0.66 (0.591-0.697)	0.658 (0.634-0.71)	0.652 (0.623-0.711)
GBM	0.685 (0.621-0.719)	0.652 (0.58-0.674)	0.641 (0.599-0.69)	0.649 (0.585-0.689)
NN	0.676 (0.62-0.702)	0.703 (0.633-0.725)	0.713 (0.632-0.722)	0.727 (0.668-0.75)
